# Utility of modified vascular corrosion casting technique in the diagnosis of fetal ductus arteriosus abnormalities

**DOI:** 10.1038/s41598-020-69694-5

**Published:** 2020-08-04

**Authors:** Yu Wang, Jiaqi Zhang, He Zeng, Haiyan Cao, Ziyi Si, Wei Feng, Mingxing Xie

**Affiliations:** 1 Department of Ultrasound, Xiangyang No.1 People’s Hospital, Hubei University of Medicine, 15 Jiefang Road, Xiangyang, 441000 China; 2 Graduate Student Training School, Xiangyang No.1 People’s Hospital, Jinzhou Medical University, Xiangyang, 441000 China; 3 Department of Ultrasound, Union Hospital, Tongji Medical College, Huazhong University of Science and Technology, 1277 Jiefang Avenue, Wuhan, 430022 China

**Keywords:** Cardiology, Heart development

## Abstract

The anatomy of ductus arteriosus (DA) can be varied in different congenital heart defects (CHDs), and it is difficult to fully discover the DA and other associated cardiac anomalies by prenatal ultrasound. This study was aimed to use the modified vascular corrosion casting technique to prepare fetal cardiovascular casts with DA anomalies, assess the casting effectiveness in evaluating the great vessels of the fetal heart and investigate the utility of cardiovascular casting for the demonstration of fetal DA abnormalities. This retrospective study enrolled fourteen fetuses (23 to 28^+2^ gestational weeks) with severe CHDs diagnosed by prenatal echocardiography and casting technique from January 2013 to July 2019. The sonographic features of DAs were carefully observed and other associated cardiovascular anomalies were also evaluated during the screening. The architectures of DAs and the whole cardiovascular system were observed and analyzed, and then the cast findings were compared with prenatal ultrasonic results. In fourteen cases, 18 ductal abnormities were indicated by prenatal echocardiography in fourteen cases, while 25 were revealed by casting. Cast findings included 4 cases of ductal stenosis, 1 case of ductal dilation, 6 cases of ductal circuity, 3 cases of right-sided ductus, 5 cases of anomalous ductal connection, 1 case of bilateral ductus and 5 cases of absent ductus. Cast findings consisted with ultrasound in 10 ductal abnormalities, revealed additional 15 ductal abnormalities miss-diagnosed by sonography, and corrected 6 abnormalities misdiagnosed prenatally. Meanwhile, 3 ductal abnormalities (reversed flow) could not be demonstrated by casts but only by ultrasound. Cast models can visually display the anatomical characteristics of ductus arteriosus, and could be successfully used in the demonstration of ductus abnormalities in fetuses with severe CHDs. Comparing with ultrasound, casting technique has its own superiority in exhibiting ductus abnormalities, especially in certain types such as course, origin and absence abnormalities of ductus.

## Introduction

Nowadays, fetal cardiac screening has been paid more and more attention^1^. As an important physiological channel for fetal circulation, ductus arteriosus, a normal structure connecting pulmonary trunk (PT) with descending aorta (DAo), is playing an indispensable role in fetal cardiovascular system^2,3^. Fetal congenital heart defects (CHDs) are often accompanied by ductal abnormalities. In these cases, the connection, course and dimension of the arterial duct can be varied^2–4^. Sometimes it is difficult to fully discover the ductal and other associated cardiac anomalies by prenatal ultrasound. Vascular corrosion casting technique is capable of accurately exhibiting the three-dimensional (3D) architecture of cardiovascular system. Our modified cast models can visually display the origin, course, dimension and relative spatial position of the great vessels, and may become an important supplement to prenatal echocardiography^5,6^. This study was aimed to introduce the modified method of preparation of fetal cardiovascular casts and assess casting effectiveness in evaluating the great vessels of the fetal heart, Meanwhile, utility of modified cardiovascular casting for the demonstration of fetal ductal abnormalities.

## Materials and methods

### Subjects

From January 2013 to July 2019, this retrospective study screened all fetuses suspected with severe CHDs induced labor in our hospital. Fourteen cases with abnormal ductus arteriosus demonstrated by casting technique were enrolled as subjects. The gestational age of fourteen fetuses ranged from 23 to 28^+^^2^ (mean 24.8 ± 1.6) weeks, and the maternal age was between 22 and 36 (mean 28.5 ± 4.0) years. This study was approved by the Medical Ethics Committee of Xiangyang No.1 People's Hospital, Hubei University of Medicine. All families provided informed consents. All methods were performed in accordance with the relevant guidelines and regulations of the Medical Ethics Committee of Xiangyang No.1 People's Hospital, Hubei University of Medicine.

### Echocardiography

Fetal echocardiographic examination was performed using an Voluson E8 or 730 (GE Medical Systems, Zipf, Austria) ultrasound machine equipped with C4-8, C4-8D and RM6C transducers (probe frequency was 4.0 ~ 8.0 MHz), according to the guidelines recommended by the International Society of Ultrasound in Obstetrics and Gynecology (ISUOG)^7^ and American Society of Echocardiography (ASE)^8^. The following standard views were included in the examination: transverse view of upper abdomen, four-chamber view, left and right ventricular outflow tract view, three-vessel view, three-vessel and trachea view, caval long-axis view, ductal and aortic arch view. The diameter of the great vessels of the heart of all cast specimens including (DA, AAo, DAo, MPA, LPA, RPA, SVC, IVC) was measured with fetal echocardiography three times.

When the fetus was found to have severe CHD, the sonographic features of ductus were carefully observed to determine the presence of ductal abnormality or not. Other associated cardiovascular anomalies were also evaluated during the examination.

### Modified cardiovascular casting

Fourteen pregnancies underwent induced labor with informed consents. In fourteen cases, nine fetuses died in utero (2 cases of placenta implantation, 2 cases of maternal bacterial infection, 2 cases of anemia in pregnancy, 3 cases of fetal intrauterine asphyxia), and the other five fetuses were terminated because of complicated intracardiac and/or extracardiac malformations. Fetal specimens were donated to our hospital for further autopsy and cast model fabrication, according to the steps as follows: (1) Thorax was cut open along the median line and bilateral costal margins with an inverted Y-shaped incision, to expose the heart, thymus, lungs and diaphragm. Peeled off the thymus, opened the pericardium, and carefully observed the structure and course of the great vessels in the chest. Midline abdominal incision was made to observe the morphology of the organs and great vessels in abdominal cavity. (2) The umbilical vessels were separated, with umbilical vein intubated with a plastic catheter and the left side umbilical artery cut-off. Acetone was injected into the umbilical vein to expel the blood clots in the cardiovascular system. (3) Casting material, a mixture composed of 10 g acrylonitrile butadiene-styrene plastic (ABS), 100 ML acetone and 2 g dyestuff, was perfused slowly into the specimen at appropriate pressure. The total amount of casting fluid was controlled between 30 to 60 ML according to the specimen’s weight, and perfusion time was controlled in approximately 30 min. (4) After solidification of the casting material, the specimen was soaked in strong acid for about one week. (5) After corrosion, the specimen was immersed in clean water and carefully rinsed. Every cast specimen was preserved in a specimen box for further observation and evaluation.

The diameters of the major vessels of the heart of all cast specimens was measured with vernier caliper three times.

### Ethical approval

This study was approved by the Medical Ethics Committee of Xiangyang No.1 People's Hospital, Hubei University of Medicine.

### Informed Consent

All families enrolled consented this study and provided informed consents.

## Results

In fourteen cases, the diameters of the great vessels were measured by casting and compared with that measured by fetal echocardiography. The results showed that there were no significant differences in the diameter of the major blood vessels between casting groups and fetal echocardiography groups (Table 1). 18 ductal abnormities were detected in prenatal echocardiography, while 25 found in cardiovascular casts (Table 2, 3). Cast findings were consistent with ultrasound in 10 ductal abnormalities. However, cast models revealed additional 15 ductal abnormalities which were missed in sonography, and corrected 6 abnormalities which were misdiagnosed prenatally. Meanwhile, 3 ductal abnormalities could not be demonstrated by casts but only by ultrasound (Table 3).

**Table 1:** Casting effectiveness evaluation in great vessels of the fetal heart.

Great vessels of the heart	Vessel diameter ((x ± *s*)(mm)		*P* value^△^
	Casting group	Echocardiography group	
DA	1.30 ± 1.47	1.37 ± 1.36	0.847
AAo	4.43 ± 1.30	4.28 ± 1.20	0.773
DAo	4.61 ± 0.73	4.36 ± 0.36	0.304
MPA	3.49 ± 1.63	3.37 ± 1.48	0.843
LPA	2.20 ± 0.49	2.98 ± 0.46	0.244
RPA	2.14 ± 0.58	2.11 ± 0.37	0.896
SVC	3.21 ± 0.51	3.11 ± 0.27	0.569
IVC	3.89 ± 0.47	3.84 ± 0.42	0.744

DA, ductus arteriosus; AAo, ascending aorta; DAo, descending aorta; MPA, main pulmonary artery; LPA, left pulmonary artery; RPA, Right pulmonary artery; SVC, superior vena cava; IVC, inferior vena cava. *P value (calculated by student *t*-test) of < 0.05 was considered statistically significant.

^△^Comparison between casting group and echocardiography group (calculated by student *t*-test).

**Table 2:** Prenatal sonographic and cast findings of fourteen fetuses with severe CHDs accompanied with ductal abnormalities.

Case	Fetal gender	Maternal age(year)	Gestational age(week)	DA abnormalities		Other associated cardiac anomalies	
				Sonographic findings	Cast findings	Sonographic findings	Cast findings
1	FEMALE	30	23	RDA	RDA, dilated DA	VSD, DORV, CoA, RAA	SV, DORV, CoA
2	female	35	26	Reversed flow in DA	Narrowed, tortuous DA	HLHS, VSD, DORV, PS, TVR	HLHS, VSD, DORV, PA
3	Female	28	23 + 2	Narrowed DA and reversed flow, anomalous DA connection (RPA-RSA)	Tortuous DA, RDA, anomalous DA connection (RPA-RSA)	Dextroversion, ECD, DORV, PS, DSVC, common trunk of LSA and LCCA	Dextroversion, VSD, DORV, PS, DSVC
4	Male	25	25 + 6	Tortuous DA, anomalous DA connection (LPA-LSA)	Tortuous DA, anomalous DA connection (LPA-LSA)	VSD, DORV, PS	DORV, PS
5	Female	28	25	Reversed flow in DA	Narrowed, tortuous DA	PS, TVR	PA
6	Female	22	25	Anomalous DA connection (LPA-LSA)	Narrowed, tortuous DA, anomalous DA connection (LPA-LSA)	SV, SA, APVC, DOV, PS	SV, SA, APVC, DOV, PS
7	Male	24	23 + 4	Dilated DA	Bilateral DA, anomalous DA connection (LPA-LDA-LSA, RPA-RDA-DAo)	SA, HLHS, DORV, AVS, CoA, DSVC	SV, SA, HLHS, DORV, IAA, DSVC
8	Female	29	25	Absent DA?	Absent DA	VSD, DORV, PS	DORV, PS
9	Female	32	23 + 3	RDA, narrowed DA	Absent DA	VSD, DORV, PVS, PS, ALSA	VSD, DORV, PVS, PS, ALSA
10	Male	27	23	Absent DA?	Absent DA	dextroversion, VSD, DORV, PS, DSVC	dextroversion, VSD, DORV, PS, DSVC
11	Male	30	24	**/**	absent DA	VSD, DORV, PS	VSD, DORV, PS
12	Male	25	27	**/**	absent DA	DORV, PS	DORV, PS
13	Male	36	25 + 2	RDA, narrowed DA , tortuous DA	RDA, narrowed DA , tortuous DA	SV, APVC, DORV, PS	SV, APVC, DORV, PS
14	Female	28	28 + 2	Absent DA?	Anomalous DA connection (LPA-DAo)	dextroversion, SV, PS, DOV	dextroversion, SV, PS, DOV

DA, ductus arteriosus; VSD, ventricle spetal defect; DORV, double-outlet right ventricle; CoA, coarctation of aorta; RAA, right aortic arch; RDA, right-sided ductus arteriosus; SV, single ventricle; HLHS, hypoplastic left heart syndrome; PS, pulmonary stenosis; TVR, tricuspid valve regurgitation; PA, pulmonary atresia; ECD, endocardial cushion defect; LSA, left subclavian artery; LCCA, left common carotid artery; DSVC, double superior vena cava; APVC, anomalous pulmonary venous connection; SV, single ventricle; SA, single atrium; DOV, double-outlet ventricle; AVS, aortic valve stenosis; IAA, interrupted aortic arch; DAo, descending aorta; PVS, pulmonary valve stenosis; ALSA, aberrant left subclavian artery. check ms table caption abb.

**Table 3:** The comparison of prenatal echocardiography and cast models in the diagnosis of fetuses with ductal abnormalities.

	Sonographic findings	Cast findings	Cast consistent with US	Additional findings in casts	Corrected by casts	Undetected in casts
**Dimension abnormality**						
Ductal stenosis	3	4	1	3	2	0
Ductal dilation	1	1	0	1	1	0
**Course abnormality**						
Tortuous ductus	2	6	2	4	0	0
Right-sdied ductus	3	3	2	1	1	0
**Origin abnormality**						
Anomalous ductal connection	3	5	3	2	10	
Bilateral ductus	0	1	0	1	0	0
**Absence abnormality**						
Absent ductus	3	5	2	3	1	0
**Flow abnormality**						
Reversed flow in ductus	3	0	0	0	03	
Total	18	25	10	15	6	3

**Figure 1:**
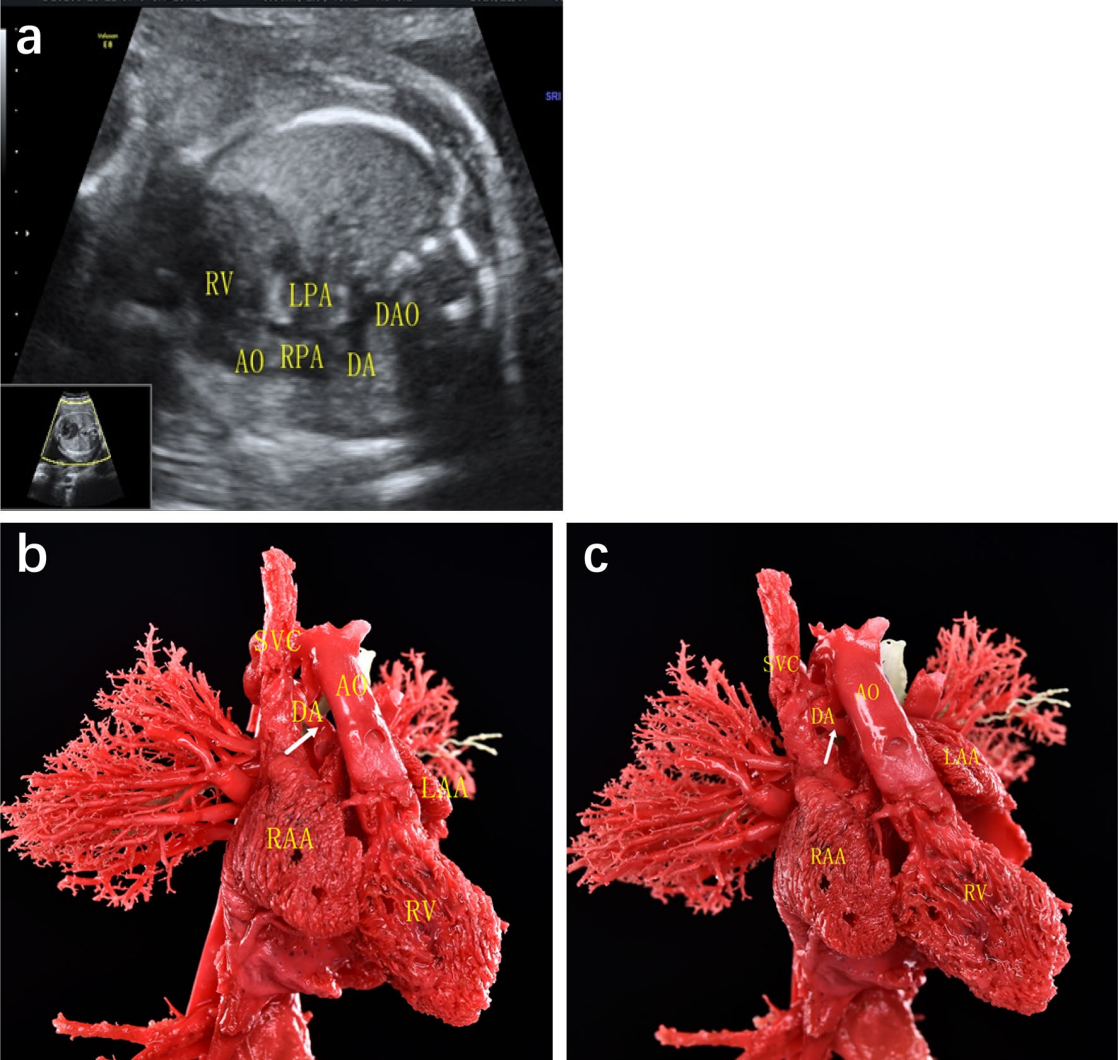
Prenatal echocardiographic and cast demonstration of a fetus with right-sided ductus arteriosus (Case 13, Table 2). (**a**) A tortuous right-sided ductus was detected by ultrasound, which connecting right pulmonary with descending aorta. (**b**), (**c**) Cast specimen also showed a tortuous ductus (arrow) connecting to descending aorta (right anterior 45° view). DA, ductus arteriosus; AO, aorta; DAO: descending aorta; LPA, left pulmonary artery; RPA, right pulmonary artery; RV, right ventricle; RAA, right atrial appendage; LAA, left atrial appendage; SVC, superior vena cava.

**Figure 2:**
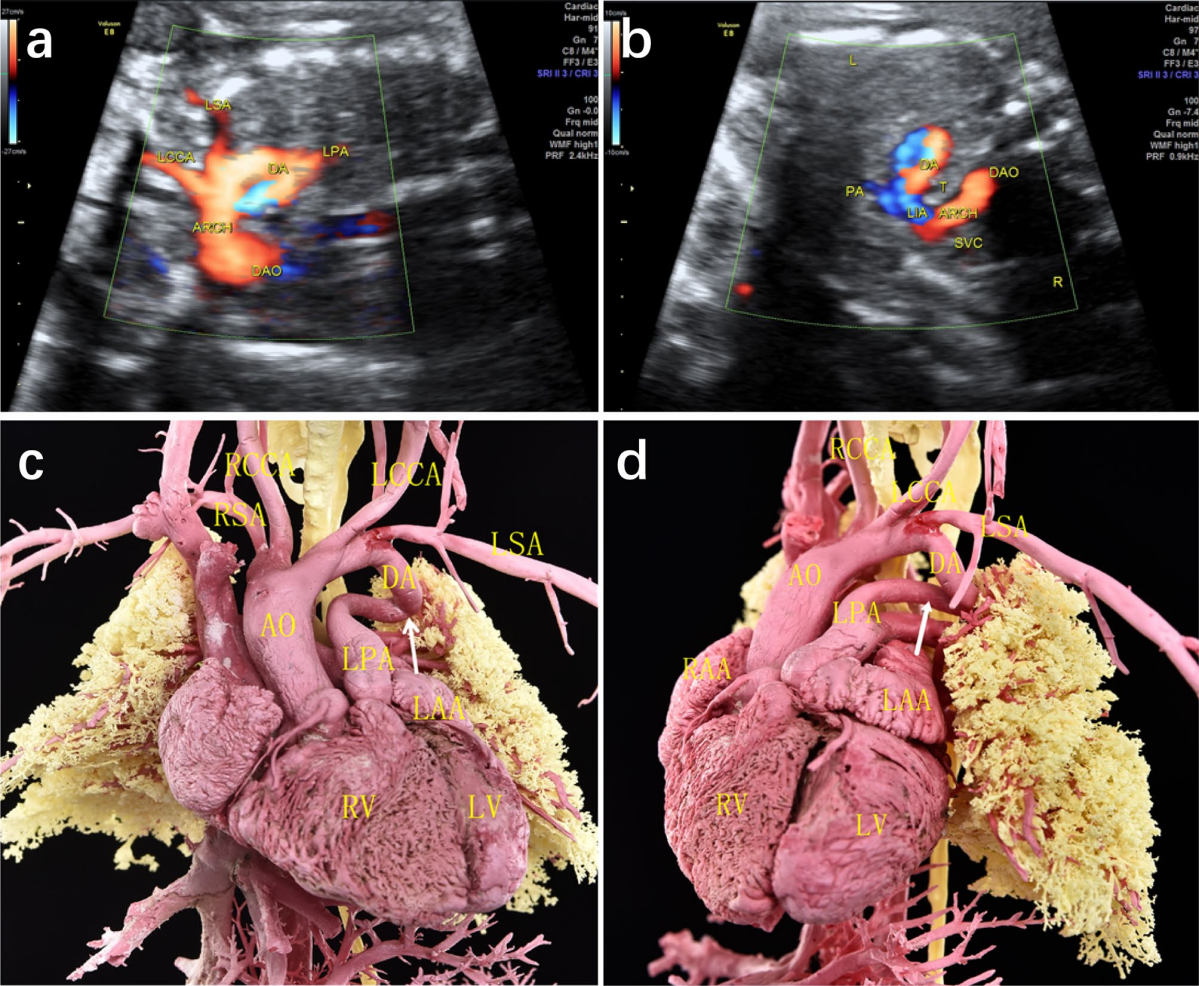
Prenatal echocardiographic and cast demonstration of a fetus with anomalous ductus arteriosus connection (Case 4, Table 2). (**a**), (**b**) Ultrasound showed ductus was connected between left pulmonary artery (LPA) and left subclavian artery (LSA). (**c**), (**d**) Cast specimen confirmed the abnormal connection of ductus (arrow), which connecting LPA and LSA. DA, ductus arteriosus; AO, aorta; ARCH, aortic arch; DAO: descending aorta; LPA, left pulmonary artery; RPA, right pulmonary artery; LSA, eft subclavian artery; RSA, right subclavian artery; LCCA, left common carotid artery; RCCA, right common carotid artery; LV, left ventricle; RV, right ventricle; LAA, left atrial appendage; RAA, right atrial appendage.

**Figure 3:**
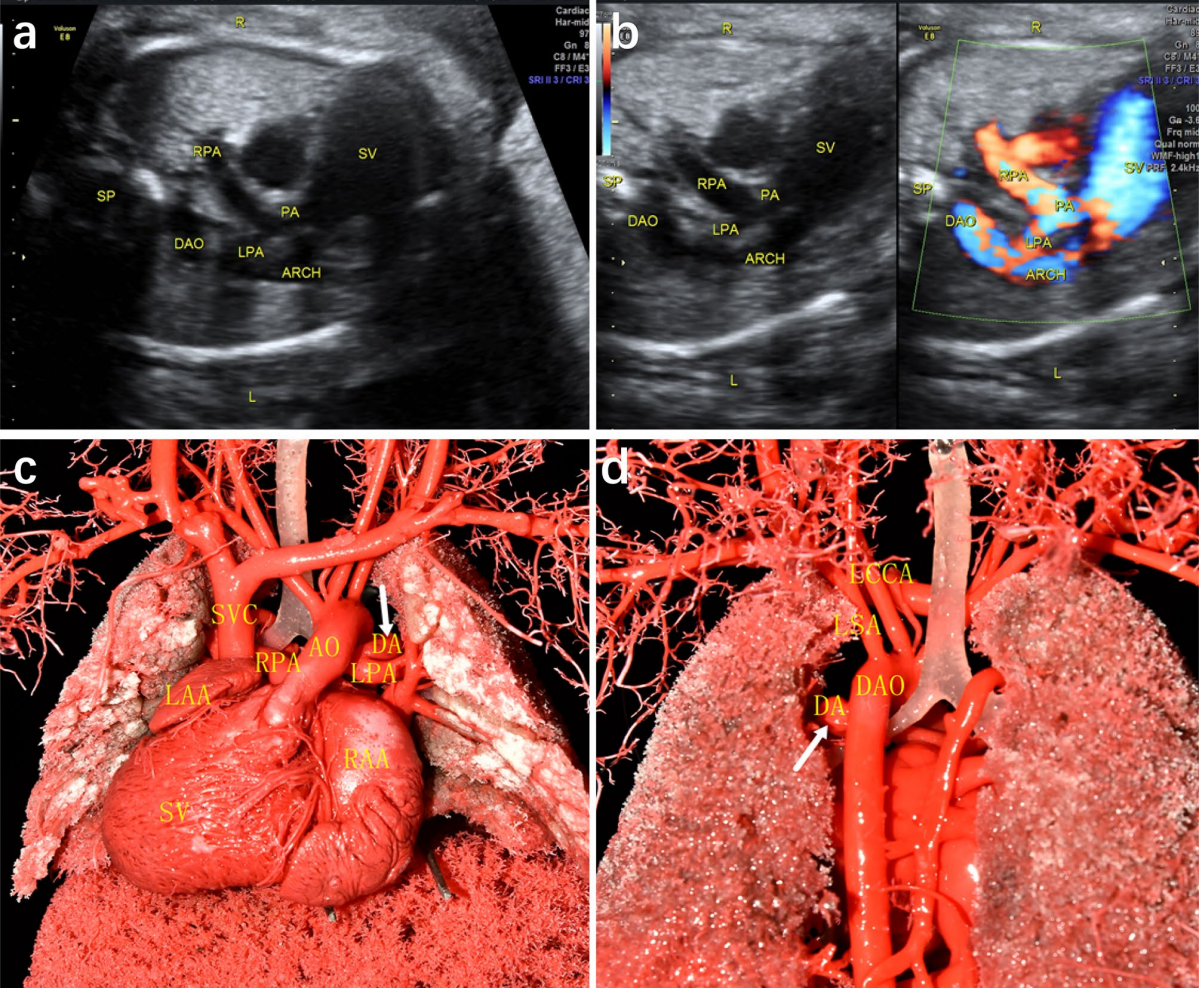
Prenatal echocardiographic and cast demonstration (Case 14, Table 2). (**a**), (**b**)Ductus was not detected by ultrasound. (**c**), (**d**) Cast specimen demonstrated the abnormal connection of ductus (arrow), which connecting LPA and DAO. DA, ductus arteriosus; AO, aorta; ARCH, aortic arch; DAO: descending aorta; LPA, left pulmonary artery; RPA, right pulmonary artery; LSA, left subclavian artery; RSA, right subclavian artery; LCCA, left common carotid artery; RCCA, right common carotid artery; SV, single ventricle; LAA, left atrial appendage; RAA, right atrial appendage.

DA abnormalities included the following different types: (1) Ductal dimensional abnormality (including ductal stenosis and ductal dilation): five cases of ductal dimensional abnormality were found by casting, including 4 cases of ductal stenosis and 1 case of ductal dilation. One case of ductal stenosis was demonstrated both by ultrasound and cast, 2 cases with narrowed DA indicated by ultrasound were not confirmed by casts, while additional 3 cases revealed slim ductus in casts. One case demonstrated dilated ductus in cast, and another case of ductal dilation suspected by echocardiography was found to be bilateral ductus by cast. (2) Ductal course abnormality (including tortuous ductus and right-sided ductus): six cases of ductal circuity were exhibited in casts, in which only 2 cases were indicated by ultrasound. Three cases of right-sided ductus arteriosus were found by echocardiography, one of them diagnosis both in ultrasound and cast (Fig. 1), one of them was corrected as absent DA by cast. And another case exhibiting right-sided ductus in cast was not detected by ultrasound. (3) Ductal origin abnormality (including anomalous ductal connection and bilateral ductus): three cases of anomalous ductal connection were detected both by ultrasound and casts (Fig. 2), and 2 additional cases were found in casts. Cast confirmed a case of bilateral ductus, which was missed in prenatal ultrasound (Fig. 3). (4) Ductal absence abnormality (absent ductus arteriosus): two cases prenatally suspected with absent ductus were confirmed by cast models, and another three cases with ultrasonic misdiagnosis or missed diagnosis were detected in casts. It is unusual that cast model revealed ductus connecting between left pulmonary artery and descending aorta in case 14, and corrected the suspicion of absent ductal by ultrasound. (5) Ductal flow abnormality (reversed flow in arterial duct): three cases showed reversed blood flow in ductus by color Doppler flow imaging (CDFI), which were not able to revealed by casts apparently.

## Discussion

Ductus arteriosus is one of the main physiological shunts in fetal circulation, and it normally maintains open before birth. The vast majority of right ventricular output is carried by arterial duct from pulmonary trunk (PT) to descending aorta (DAo), leaving only a small amount of blood flowing to the lungs^9^. Ductus can help to maintain a stable cardiac output (CO), even in fetuses with severe CHDs^2,3^. In recent years, several studies showed a variety of fetal CHDs were always associated with different ductal abnormalities4. Moreover, certain types of CHDs could even influence the ductal morphology and hemodynamics^2–4^. In these cases, sonographic features of ductus arteriosus could be diverse, which makes it hard to diagnose accurately in utero. Furthermore, prenatal echocardiography may often be affected by a series of factors, such as fetal position and gestational weeks, amniotic fluid volume, maternal abdominal wall thickness, ultrasonic resolution, etc. Hence, there are still some limitations in prenatal diagnosis of fetal CHDs by using ultrasound^10^.

In this study, we investigated the utility of vascular corrosion casting technique to assess casting effectiveness in evaluating the major blood vessels of the fetal heart and reveal the real anatomy of ductus arteriosus in different CHDs^11^. Cardiovascular casting is a method of injecting casting material into the vessels, corroding the soft tissues by strong acid, and leaving the real architectures of cardiovascular system^12^. This method can display the three-dimensional (3D) morphology of fetal severe CHDs. Its unique advantages will help to improve the understanding of fetal cardiac anatomy and the accuracy of prenatal diagnosis^5,6^. This study indicated cast models could vividly reveal the origin, course, dimension and spatial relationship of ductus arteriosus and other great vessels.

Our study showed various ductal abnormalities were presented in severe CHDs fetuses. By comparing cast with ultrasound, we found more ductal abnormalities could be detected in cast models (25 vs. 18). Casts were not only helpful to confirm prenatal ultrasound results, but also beneficial to correct the missed diagnosis and misdiagnosis by echocardiography (Figs. 1, 2, 3). While, on the other hand, ultrasound also had its special advantages^13^. For instance, hemodynamic changes in ductus can only be displayed by color Doppler. As we know, blood in ductus arteriosus flows from the main pulmonary trunk to the descending aorta in normal fetus. However, some CHDs may change the hemodynamics. In our study, case 2, 3, 5 all demonstrated reversed flow across DA (from descending aorta to pulmonary artery), and were all associated with pulmonary atresia or pulmonary stenosis.

By using ultrasound combined with cast models, we found several different types of ductal abnormalities in these fourteen cases, which could be divided into dimension abnormality (DA stenosis and dilation), course abnormality (tortuous DA, right-sided DA), origin abnormality (anomalous DA connection, bilateral DA), absence abnormality (absent DA) and flow abnormality (reversed flow in DA). Eight cases had more than one abnormality. These abnormalities are always associated with complicated CHDs. And some of these abnormalities (such as bilateral ductus arteriosus) are rarely seen. Moreover, the existence or absence of ductus arteriosus may change fetal prognosis and therapeutic strategy. Therefore, accurate prenatal diagnosis of ductus arteriosus abnormalities could help perinatal management. In our study, casting technique has its own superiority in revealing these ductal abnormalities, especially in the demonstration of abnormal course, origin and absence of ductus arteriosus. It will help the sonographers gain more insight into the real anatomy of complicated CHDs. Ductal circuity was found in six cases by casts, while only two cases were detected prenatally. Absent duct was suspected in three cases by ultrasound, while casts finally confirmed five cases with absent DA. Anomalous ductus connection was relatively rare, and we found five cases in cast models, with only three cases noticed by ultrasound. Moreover, as an unusual anomaly, one case of bilateral ductus arteriosus was detected by casting technique, which was missed in utero. In this case, the left-sided ductus was connected between left pulmonary artery (LPA) and left subclavian artery (LSA), while the right-sided ductus connected right pulmonary artery (RPA) and descending aorta (DAo). As we know, ductus arteriosus embryologically originates from the distal part of the left sixth arch, and the right sixth arch degenerates eventually^14^. So bilateral DAs might be caused by failed degeneration of bilateral sixth aortic arch. Bilateral ductus is always associated with complicated cardiac anomalies in previous literatures^15,16^. And in this case, we also found this fetus had a series of other cardiac malformations, such as single atrium (SA), hypoplastic left heart syndrome (HLHS), double-outlet right ventricle (DORV), aortic stenosis (AS), interrupted aortic arch (IAA)and double superior vena cava (DSVC).

Casting and ultrasound both have their own superiorities. Ultrasound is able to display hemodynamic changes by color Doppler, while casting could not. So, the flow direction changes in ductus arteriosus will certainly be missed on cast specimens. On the other hand, casting can demonstrate the real 3D architecture of cardiovascular system, while ultrasound usually display sonographic anatomy in 2D planes. Some abnormalities, such as anomalous ductal connection and bilateral ductus, the course and origin of ductus arteriosus is hardly to demonstrate in single plane by 2D ultrasound. 3D ultrasound may help to reveal these ductal abnormalities, but in fact, these vessels are always too tiny or too curved to be clearly observed or demonstrated.

## Conclusion

We successfully applied the modified vascular corrosion technique to prepare fetal cardiovascular cast models. Cardiovascular cast models can not only effectively show the diameter of the major blood vessels of the fetal heart, but also visually display the anatomical characteristics of ductus arteriosus, and could be successfully used in the demonstration of ductus abnormalities in fetuses with severe CHDs^17–19^. Comparing with ultrasound, casting technique has its own superiority in exhibiting ductus abnormalities, especially in certain types such as course, origin and absence abnormalities of ductus. Casting is of great value for sonographers and clinicians to better understanding the spatially anatomy of fetal ductus and even the whole cardiovascular system, and is helpful to improve the diagnostic capability of prenatal echocardiography^20^.
